# Predicting change trajectories of neuroticism from baseline brain structure using whole brain analyses and latent growth curve models in adolescents

**DOI:** 10.1038/s41598-020-58128-x

**Published:** 2020-01-27

**Authors:** Simone Kühn, Anna Mascherek, Tobias Banaschewski, Arun L. W. Bokde, Christian Büchel, Erin Burke Quinlan, Sylvane Desrivières, Herta Flor, Antoine Grigis, Hugh Garavan, Penny Gowland, Andreas Heinz, Bernd Ittermann, Jean-Luc Martinot, Marie-Laure Paillère Martinot, Frauke Nees, Dimitri Papadopoulos Orfanos, Tomáš Paus, Luise Poustka, Sabina Millenet, Juliane H. Fröhner, Michael N. Smolka, Henrik Walter, Robert Whelan, Gunter Schumann, Ulman Lindenberger, Jürgen Gallinat

**Affiliations:** 1 University Medical Center Hamburg-Eppendorf, Department of Psychiatry and Psychotherapy, Martinistrasse 52, 20246 Hamburg, Germany; 2 Lise Meitner Group for Environmental Neuroscience, Max Planck Institute for Human Development, Berlin, Germany; 3 Department of Child and Adolescent Psychiatry and Psychotherapy, Central Institute of Mental Health, Medical Faculty Mannheim, Heidelberg University, Square J5, 68159 Mannheim, Germany; 4 Discipline of Psychiatry, School of Medicine and Trinity College Institute of Neuroscience, Trinity College Dublin, Dublin, Ireland; 5 University Medical Centre Hamburg-Eppendorf, House W34, 3.OG, Martinistr. 52, 20246 Hamburg, Germany; 6 Medical Research Council - Social, Genetic and Developmental Psychiatry Centre, Institute of Psychiatry, Psychology & Neuroscience, King’s College London, London, United Kingdom; 7 Department of Cognitive and Clinical Neuroscience, Central Institute of Mental Health, Medical Faculty Mannheim, Heidelberg University, Square J5, Mannheim, Germany; 8 Department of Psychology, School of Social Sciences, University of Mannheim, 68131 Mannheim, Germany; 9 NeuroSpin, CEA, Université Paris-Saclay, F-91191, Gif-sur-Yvette, France; 10 Departments of Psychiatry and Psychology, University of Vermont, 05405 Burlington Vermont, USA; 11 Sir Peter Mansfield Imaging Centre School of Physics and Astronomy, University of Nottingham, University Park, Nottingham, United Kingdom; 12 Department of Psychiatry and Psychotherapy, Campus Charité Mitte, Charité, Universitätsmedizin Berlin, Charitéplatz 1, Berlin, Germany; 13 Physikalisch-Technische Bundesanstalt (PTB), Braunschweig and Berlin, Germany; 14 Institut National de la Santé et de la Recherche Médicale, INSERM Unit 1000 “Neuroimaging & Psychiatry”, University Paris Sud, University Paris Descartes - Sorbonne Paris Cité; and Maison de Solenn, Paris, France; 15 Institut National de la Santé et de la Recherche Médicale, INSERM Unit 1000 “Neuroimaging & Psychiatry”, University Paris Sud, University Paris Descartes - Sorbonne Paris Cité; and AP-HP, Department of Adolescent Psychopathology and Medicine, Maison de Solenn, Cochin Hospital, Paris, France; 16 Bloorview Research Institute, Holland Bloorview Kids Rehabilitation Hospital and Departments of Psychology and Psychiatry, University of Toronto, Toronto, Ontario M6A 2E1 Canada; 17 Department of Child and Adolescent Psychiatry and Psychotherapy, University Medical Centre Göttingen, von-Siebold-Str. 5, 37075 Göttingen, Germany; 18 Clinic for Child and Adolescent Psychiatry, Medical University of Vienna, Währinger Gürtel 18-20, 1090 Vienna, Austria; 19 Department of Psychiatry and Neuroimaging Center, Technische Universität Dresden, Dresden, Germany; 20 School of Psychology and Global Brain Health Institute, Trinity College Dublin, Dublin, Ireland; 21 Max Planck UCL Centre for Computational Psychiatry and Ageing Research, London, United Kingdom

**Keywords:** Development of the nervous system, Agency

## Abstract

Adolescence is a vulnerable time for personality development. Especially neuroticism with its link to the development of psychopathology is of interest concerning influential factors. The present study exploratorily investigates neuroanatomical signatures for developmental trajectories of neuroticism based on a voxel-wise whole-brain structural equation modelling framework. In 1,814 healthy adolescents of the IMAGEN sample, the NEO-FFI was acquired at three measurement occasions across five years. Based on a partial measurement invariance second-order latent growth curve model we conducted whole-brain analyses on structural MRI data at age 14 years, predicting change in neuroticism over time. We observed that a reduced volume in the pituitary gland was associated with the slope of neuroticism over time. However, no relations with prefrontal areas emerged. Both findings are discussed against the background of possible genetic and social influences that may account for this result.

## Introduction

The search for neural correlates as underlying foundation of personality has received reasonable attention in the field of personality neuroscience, where traits as one core aspect of personality are linked to underlying brain areas and function in order to understand personality and individual differences on ‘biological’ level^1^. The Five Factor Model of personality^2,3^ has generally been applied as theoretical taxonomy, comprising Neuroticism (i.e., being emotionally unstable, worried, and negative), Extraversion (i.e., being assertive), Openness to experience (reflecting a curiosity in novelty and distraction), Conscientiousness (i.e., being self-organized), and Agreeableness (i.e., being cooperative) as main traits. Although the Five Factor Model is historically as well as currently only one of many, it remains the most influential, ubiquitous, and most often applied theory in the scientific discourse. Broad consensus across theories can be found for Extraversion and Neuroticism, which are basically present in all theories, though differing in detail and exact labeling^4–7^. Of the five personality traits, neuroticism has been most intensively studied and is linked most clearly to brain structure^8–11^. It is also most consistently linked to psychopathology, especially depression and anxiety, but also to the development of posttraumatic stress disorder (PTSD)^12^. Different brain structural measures have been linked to personality, such as the volume of specific regions and cortical thickness^9^. Also, endocrinological aspects such as the relation between personality and cortisol levels in neurotic individuals have been evaluated^13^.

Neuroticism changes across the lifespan, with adolescence as a vulnerable time of developmental change. In general, results point towards maturation with neuroticism peaking in early adulthood and decreasing from then^2,14^. Depending on selected samples, different age profiles are described, despite the general similarity in shape of trajectories^15–17^. This is attributable to interindividual differences that are common in personality development^18^.

During adolescence multiple endocrinological changes occur, influencing behavioral, physical, emotional, and brain structural changes^19,20^. For neuroticism, the hypothalamic–pituitary–adrenal (HPA) axis activity and cortisol-levels as a proxy for HPA-activity, have been focused on. The HPA axis coordinates stress-reactions in an organism. The interplay of hypothalamus, pituitary gland, and adrenal gland (together forming the HPA axis) maintains the secretion of adrenocorticotropic hormone (ACTH) and cortisol, which regulate the system’s reaction to stress. A body of research shows that higher levels of neuroticism are associated with lower levels of cortisol^13,21–23^. However, also contradictory results emerge^24^ which may, not least, be due to the difficulty to assess cortisol level without confounders. Studies on the relation between brain structural aspects of the HPA-axis and its function are scarce to date.

Turning to neural correlates, adolescence is likewise a vulnerable time for brain structural development. With the onset of puberty, hormones drive changes in white and grey matter, most often measured by means of volume, size of surface area, and cortical thickness^19,20^. Although there is no general answer to the developmental trajectories of grey and white matter, it might be best summarized that development of cortical grey matter follows a nonlinear trajectory increasing across childhood, reaching a peak in adolescence and declining into early adulthood (cortical thinning) with lobe-specific differentiation^25,26^. For white matter integrity, increases across childhood and adolescence have been found until the 3^rd^ or 4^th^ decade followed by decline, hence, also generally following a nonlinear trajectory but delayed in time^19,25,27–29^. Neuroticism has been associated with reduced rates of cortical thinning. Ferschmann, *et al*.^30^ speculated that individuals higher in neuroticism might demonstrate slower rates of cortical maturation, that is, exhibit a thicker cortex compared to less neurotic individuals. In their study in a sample of participants aged 8–19 years, emotional stability was related to cortical thinning in the right superior temporal cortex, as well as in different prefrontal regions. Their study corroborates results from Riccelli, *et al*.^31^, but not Holmes, *et al*.^32^. A meta-analysis by Mincic^9^ on neuroanatomical correlates of negative emotionality-related traits showed that, most consistently, neuroticism was associated with reduced grey matter in prefrontal areas, especially in the medial and lateral orbitofrontal cortex. Also, reduced grey matter was reported in the parietal cortex, whereas results for motor and premotor areas were mixed. Mixed results were also reported for subcortical structures, however, connections to the amygdala and hippocampal areas were reported. Abram and DeYoung^8^ summarized current knowledge concerning neural correlates of neuroticism similarly. Structural and functional magnetic resonance imaging (MRI)-studies repeatedly show relations between neuroticism and regions within the prefrontal cortex and its connections to the amygdala. This region is involved in emotion-processing and the development of mood-disorders^33^.

In the present study we investigate the value of voxel-based whole-brain analyses at baseline in a structural equation modelling framework for the *prediction* of longitudinal change (i.e., predicting the slope) in neuroticism in a large sample of adolescents. Methodologically, this goes beyond previous analyses, as we apply whole-brain structural data as predictor for developmental change of a self-reported measure, instead of extracted regions of interests or global brain measures (intracranial volume, white matter hyperintensities). Despite the explorative nature of our analyses (whole-brain vs ROI), we expect to find smaller volumes in prefrontal areas, amygdala, and temporal lobe in neurotic individuals. We also expect that smaller volumes will predict less decrease in neuroticism across time. We further speculate to find smaller hypothalamus and pituitary gland in individuals high in neuroticism, as this should be the structural analogue of lower cortisol levels.

## Results

### Measurement invariance

**Figure 1:**
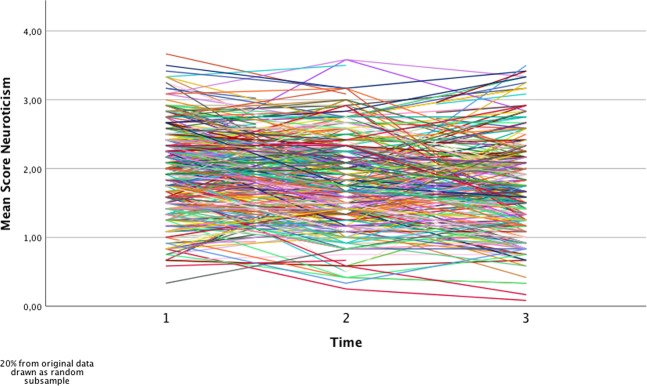
Mean scores of neuroticism across time in a random subsample of 20% of the original data. Mean-scores of neuroticism were plotted across our three measurement occasions. To improve readability, we drew a random subsample consisting of 20% of individuals from the original dataset.

As a first step, the 12 neuroticism-items of the NEO-FFI serving as manifest indicators of the measurement model were tested for different degrees of measurement invariance (see Fig. 1 for the development of mean scores). Configural invariance was tested, that is, all 12 indicators were forced to load on the one latent factor at each time-point. The model fitted the data well (adjusted *χ*^2^ = 1134.62, *df* = 534, *p* < 0.001, *RMSEA* = 0.025 (90% CI: 023–0.027), *CFI* = 0.96). In a second step, weak measurement invariance was tested, that is factor loadings were constrained to be equal across time. This model did not result in a substantial loss of model fit, although the chi-square-difference was significant (adjusted *χ*^2^ = 1,208.75, *df* = 556, *p* < 0.001, RMSEA = 0.025 (90% CI: 0.024–0.027), CFI = 0.96; Satorra-Bentler scaled [S-B] Δ*χ*^*2*^ = 76.83, *Δdf* = 22, p < 0.05; no difference in CFI and RMSEA). However, as CFI and RMSEA did not change, we accepted the weak MI-model^34^. For the strong measurement invariance model, that is constraining intercepts to be equal across time, a significant decrease in model fit emerged (adjusted *χ*^*2*^ = 1,673.23, *df* = 578, *p* < 0.001, RMSEA = 0.032 (90% CI: 0.031–0.034), CFI = 0.93; S-B Δ*χ*^*2*^ = 497.11, *Δdf* = 22, p < 0.05; ΔCFI = 0.03 and outside RMSEA CI-interval of the weak MI-model), indicating that differences in at least one intercept could not be explained solely by differences in the latent construct (neuroticism) over time. We inspected modification indices and size of residuals and, finally, decided to freely estimate 5 intercepts across time (for items “I often feel that I am not as good as others”, “I rarely feel lonely or blue”, “I rarely feel fearful or anxious”, “I often get angry at the way people treat me”, “At times I have been so ashamed I just wanted to hide”). According to Byrne, *et al*.^35^, testing for differences on factor level is reasonable with partial strong measurement invariance as long as model specification includes multiple indicators and at least one measure is invariant (see Table 1). With 7 intercepts being invariant across time in the present study, this requirement is met and the model yielded an acceptable fit (adjusted *χ*^*2*^ = 1,260.73, *df* = 568, *p* < 0.001, RMSEA = 0.026 (90% CI: 0.024–0.028), CFI = 0.96; S-B Δ*χ*^*2*^ = 54.28, *Δdf* = 10, p < 0.05; no difference in CFI and within RMSEA CI-interval of the weak MI-model).

**Table 1:** Standardized Factor loadings of the items indicating neuroticism in the NEO-FFI.

Manifest Indicators	T1	T2	T3
I am not a worrier	0.28	0.26	0.29
I often feel that Im not as good as others*	0.59	0.59	0.65
When I’m under a great deal of stress, sometimes I feel like I’m going to pieces	0.59	0.61	0.65
I rarely feel lonely or blue*	0.50	0.54	0.58
I often feel tense and jittery	0.59	0.61	0.65
Sometimes I feel completely worthless	0.71	0.72	0.78
I rarely feel fearful or anxious*	0.49	0.50	0.55
I often get angry at the way people treat me*	0.44	0.46	0.49
Too often, when things go wrong, I get discouraged and feel like giving up	0.59	0.63	0.68
I am seldom sad or depressed	0.47	0.52	0.56
I often feel helpless and want someone else to solve my problems	0.62	0.64	0.69
At times I have been so ashamed I just wanted to hide*	0.49	0.45	0.49

Note. T1: first measurement occasion (14 years of age); T2: second measurement occasion (16–17 years of age); T3: third measurement occasion (19 years of age). Factor loadings refer to the final model. All factor loadings were significant. Standardized factor loadings can be different in numbers although equality constraints do hold, because of the standardization. Items with (*) have unconstraint *intercepts* across measurement occasion according to the partly *strong* measurement invariance model.

### Structural equation modelling

**Figure 2:**
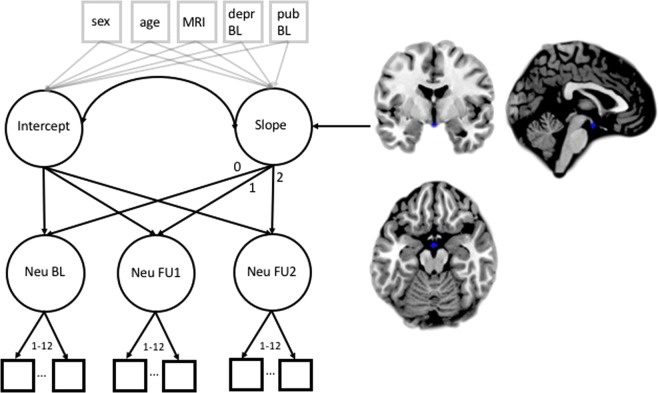
Second-order latent growth curve model. Neu = Neuroticism, depr = depression, pub = puberty according to Pubertal Development Scale, BL = baseline, FU = Follow-Up, MRI = Scanner-site. Scanner was not a single indicator as depicted for reasons of simplicity, but consisted of 8 separate indicators dummy coding the different scanners used. Nuisance variables are painted in light grey. Brain regions in blue indicate a significant regression path from brain voxel to the latent slope describing decrease in neuroticism over time (2, −4, −17, p < 0.001, cluster > 50 voxels). 1–12: Indicating that 12 manifest neuroticism-variables at each time-point were assessed, as for simplicity only two are drawn.

Analyses continued adding a second-order latent growth curve model onto the partial strong measurement invariance model with intercept and slope in order to analyze development and, finally, the predictive value of MRI-data on change trajectories in neuroticism. This model yielded an acceptable fit (adjusted *χ*^*2*^ = 1,267.43, *df* = 571, *p* < 0.001, *RMSEA* = 0.026 (90% CI: 024–0.028), *CFI* = 0.96, see Fig. 2 for details).

#### Development of neuroticism

We then added age, sex, scanner-site (dummy coded), to the model as covariates, no matter whether the regression paths were significant or not, since it is common practice in neuroimaging studies to control for these nuisance variables (see Table 2). The final model, excluding the brain variable (since this varied for each voxel of the brain) also included depression at baseline^36^ and puberty stage^37,38^ at baseline and demonstrated an acceptable model fit (adjusted *χ*^*2*^ = 2,853.82, *df* = 945, *p* < 0.001, *RMSEA* = 0.034 (90% CI: 0.032–0.035), *CFI* = 0.89). Especially the strong decrease in CFI is due to the fact, that most of the covariates did not yield significant paths, hence, from a statistical point of view do not contribute any information, but rather reduce parsimony of the model and converge towards the null-model. Attending to a reviewer, we also tested whether a latent basis model would be better suited to adjust for possible non-linear change. A model with freely estimating the second-timepoint (constraining slope loadings to 0, *, and 1) led to an estimate significantly different from zero with 0.432, which indicates an almost linear trend. According to a S-B Δ*χ*^*2-*^test, RMSEA, and CFI it did not fit the data significantly better, hence, we kept the linear model as it is more parsimonious (*χ*^*2*^ = 2,853.18, *df* = 944, *p* < 0.001, RMSEA = 0.034 (90% CI: 0.032–0.035), CFI = 0.89; S-B Δ*χ*^*2*^ = 1.15, *Δdf* = 1, ns).

**Table 2:** Standardized coefficients for time-invariant covariates of the final model.

Time-invariant covariates	intercept	slope
	standardized coefficients	standardized coefficients
Age	−0.02	0.01
Sex^§,‡^	0.60*	0.32*
Puberty Stage	0.02	−0.06
Depression	0.3*	−0.12*
**Scanner-Site**^**†**^		
Berlin^§^	−0.22	0.29
Dresden^§^	0.14	0.05
Hamburg^§^	−0.25*	0.44*
Mannheim^§^	−0.10	0.19
London^§^	0.37	0.26
Nottingham^§^	0.29*	0.26
Dublin^§^	0.12	0.74*
Paris^§^	0.31*	−0.04

Note. Covariates were assessed at first measurement occasion (T1). * denotes significant coefficients. ^§^We report the Mplus “StdY”-standardization, which is recommended for binary covariates. See Mplus User’s Guide for details on exact calculation of the coefficients. ^‡^Sex was coded with males being the reference category. ^†^The nine scanner-sites were dummy-coded, with Berlin as site having two different MRI-Scanner. One General Electric Scanner in Berlin served as reference.

Turning to the results from the growth-curve model we report significant decreases in neuroticism on mean level (negative mean of the slope), with significant inter-individual differences (significant variance). Additionally, we found significant variances for the level, indicating inter-individual differences in the initial value at baseline. We also found a negative relation between level and slope indicating that individuals higher in neuroticism at baseline tend to decrease stronger over time (see Table 3).

**Table 3:** Standardized estimates for intercept and slope of the second-order latent growth curve model.

Latent Estimates	Estimates	
	Mean (standard error)	Mean (standard error)
Intercept	3.5 (1.5)	0.8 (0.03)
Slope	−0.7 (0.18)	0.9 (0.03)
	Correlation	
Intercept-Slope	−0.2	

Note. All estimates were significant.

#### Brain volume as predictor

In a final and most important step, we conducted an exploratory whole-brain analysis on brain (grey and white matter) probability maps to predict change in neuroticism on latent level over time from each voxel. We chose exploratory analyses with conservative thresholding (p > 0.001) and clustering (n > 50 voxels) but without correction for multiple comparisons. On grey matter maps only one significant result emerged. Most interestingly, however, we found reduced volume in the pituitary gland predicting the slope of neuroticism over time (2, −4, −17, *p* < 0.001, cluster > 50 voxels). This is especially interesting, as studies linking cortisol-level with neuroticism have been conducted, however, evidence with the pituitary gland volume (PGV) as marker for neuroticism has, to the best of our knowledge, not yet been rendered. Also, being able to predict development of neuroticism from structural brain data at baseline has not been shown before. Correlations between grey matter data and the intercept were rendered non-significant after thresholding (*p* < 0.001) and cluster size correction (cluster size > 50 voxels). The same analysis on white matter probability maps did not result in any significant clusters.

## Discussion

We found a general decrease in neuroticism across time with inter-individual differences reflected by the mean and variance of the slope in the self-reported questionnaire data. Also, individuals differed in neuroticism at baseline. The negative relation between level and slope might reflect that we measured individuals at different developmental stages, although numerically same aged. The steeper decline in individuals with high levels of neuroticism at baseline might reflect an assessment before the normative decline of neuroticism in adolescence. Individuals starting lower off in our analyses might have started to decline at baseline. Our interpretation fits into existing studies reporting similar change trajectories and inter-individual differences, however, in different age-ranges^2,17^. This strengthens the interpretation that our sample was “caught” at different developmental stages despite age homogeneity.

Turning to neural correlates, we found reduced pituitary gland volume (PGV) at baseline predicting less decline of neuroticism. Previous studies linked neuroticism with low levels of cortisol, which is related to pituitary gland volume^39–41^. However, to the best of our knowledge, no studies have linked PGV and neuroticism directly. Higher pituitary gland volume (PGV) is linked to an increased number of corticotropin releasing cells, hence, increased levels of ACTH and cortisol. Seemingly, smaller pituitary volume is linked to less HPA axis activity, therefore, less cortisol. We speculate that the predictive value of the PGV could be important in the age group studied. With puberty, the organism undergoes hormonal changes and reorganization. With no relation at baseline but in the prediction of neurotic development, we speculate that PGV might be informative as an early marker for future cortisol production. The smaller PGV at age 14 yrs might negatively foreshadow the cumulative effect of hormones on neurotic development. Our interpretation is limited to the fact that no direct measures of cortisol levels were assessed in the present study and our statistical analyses were exploratory in nature. The relation between PGV and cortisol is understudied to date and inferences remain speculative. Further research is needed to shed light on the relation between cortisol level and PGV. Sex was added as covariate to the model in order to adjust for potential influences. Sex differences were significant for both, intercept and slope, indicating that females had higher levels of neuroticism at baseline and a reduced change rate across time. Sex differences were not the focus of the present study, hence, no potentially explanatory variables (such as e.g., sex hormones) were assessed. Inferences about causes driving this differential devel-opment are thus not warranted from the present data. In future studies, this aspect, however, should be addressed as explanation of different developmental trajectories would significantly add knowledge over and above the mere description of an effect.

No relation with prefrontal areas expected from the literature emerged. One might simply argue that prefrontal areas are of no *predictive* value for the development of neuroticism in adolescence. While this could be true, it seems oversimplifying as both constructs (cortex and personality) are complex. Second, plasticity of the prefrontal cortex is pronounced in puberty. Hence, possible relations could be masked by uncontrolled noise from other influences in our sample. Third, on intra-individual level, evidence exists for heterochronic development (regional specificity), of grey matter in humans, with prefrontal areas developing late^25^. We speculate that the developmental state of prefrontal areas at age 14 was not yet relevant for longitudinal change in neuroticism. Although our results are contradicted by Ferschmann, *et al*.^30^ in a sample of 74 adolescents between 8–19 yrs, our interpretation might still be valid due to the larger age range in their study.

Integrating the results above leads to major personality models that state both, genetical as well as social and environmental influences^3,42^. We speculate that our findings reflect that. Different entities might drive the development of neuroticism at different times. During puberty, development could be driven by hormonal influences that are less controllable by the individual, reflecting the genetically predisposed aspect. We speculate that this was assessed in our study and shows in the relation between development of neuroticism and PGV as a proxy for cortisol-level. A second aspect of personality development might be represented by cognitive, self-reflective processes and less genetically but socially and environmentally influenced. This should be highly individual, underlying cognitive processes and reflecting one’s individual way of life and environment. The link between neuroticism and prefrontal areas would reflect those cognitive aspects of personality development. However, as the prefrontal cortex starts to develop late, this was not captured in our sample. Indirect empirical support comes from work that has linked neuroticism to emotional dysregulation, low self-esteem, rumination^43^ and, in turn, to prefrontal areas^44^. Against the background of the present study, this interpretation remains speculative. Longitudinal studies with different age cohorts are needed to show the differential impact of PGV and prefrontal areas on the development of neuroticism and its behavioral correlate. However, the results together with hints from existing literature render our ideas interesting and deserve further consideration.

The results of the present study are based on a very large, age-homogeneous sample of adolescents. Especially in the realm of imaging-studies, this is a major strength.

A critical limitation is the lack of actual cortisol concentration. Because the relation between PGV and cortisol-level has not been established, our interpretation remains vulnerable with respect to these inferences. Future studies are needed to explicitly demonstrate that PGV may be used as a proxy for cortisol levels. In the same vein, the assessment and analyses of sex hormones could be of special interest in a sample of adolescents. We acknowledge this as a limitation of the present study and encourage future research to also include assessment of sex hormones (levels).

Second, our latent-growth curve model was based on a partial strong measurement invariance model. This is feasible from a methodological point of view^35^, however, has to be kept in mind as deviation from full strong measurement invariance, leading to less reliable interpretations of differences on mean level. Content-wise, some non-invariance might be attributable to the testing situation. Non-invariant items were possibly more vulnerable to situational effects, hence, are confounded by state.

Third, although the sample was age-homogeneous, second and third measurement occasion were de facto conducted between 16–17 yrs of age at second and 18–20 yrs of age at third measurement occasion and remain uncontrolled for in our data.

Depending on point of view, whole-brain analyses are a strength or limitation all at once. Whole-brain analyses require conservative thresholding and clustering. Hence, the report of results is conservative and we might have missed relevant relations. Nevertheless, whole-brain analyses allowed to exploratorily analyze whether (and which) brain structural areas are of predictive value for the development of neuroticism. Because ROI-analyses need prior information concerning the areas involved, they are not suited for exploratory analyses. That said, we encourage the reader to bear in mind that with a more liberal analysis, additional areas might have proved influential. Related to that, our results must be clearly seen as exploratory, however interesting they might be. Future research is needed to replicate and confirm the potential relationship between pituitary gland volume and neuroticism in adolescence. Confirmatory ROI-analyses with the pituitary gland as region of interest in an independent experimental sample would be needed before drawing definite conclusions. Until then, results of the present paper remain an interesting, yet clearly exploratory finding.

## Methods

### Participants

We used data from 1,808 healthy 14-year old adolescents (mean age = 14.4 years, SD = 0.46 years; 884 males) who were recruited within the scope of the IMAGEN project, a European multi-center genetic-neuroimaging study in adolescence^45^. The selection of the participants was based on the availability of structural imaging data at age of 14 years additionally to self-report personality data. Self-report retest data at age 16–17 years was available for 1,414 participants (mean age = 16.4 years, SD = 0.65 years; 692 males) and at age 19 years for 1,282 participants (mean age = 19.0, SD = 0.74 years; 609 males). We had three different patterns of dropout: dropout after baseline, dropout after second measurement occasion, and intermittent dropout after second measurement occasion with return for the third measurement occasion. The dropout-groups (including completers) did not differ significantly with respect to sex and depression at baseline. Originally measured in days of age, means for the respective dropout-groups were as follows: non-dropout group mean age = 5256 days, SD = 161 days; dropout after first measurement occasion: mean age = 5290 days, SD = 150 days; dropout after second measurement occasion: mean age = 5265 days, SD = 199 days; intermittent dropout after second measurement occasion with return for the third measurement occasion: mean age = 5311 days, SD = 161 days. Age differences were statistically significant between completers and dropout after first measurement occasion and intermittent dropout, respectively. Also, the difference between dropout after second measurement occasion and intermittent dropout proved statistically significant. However, all differences were interpreted as irrelevant for the study. All analyses were performed in accordance with relevant guidelines and written informed consent was obtained from all participants as well as from their legal guardians. The adolescents were recruited from secondary schools. The study was approved by the ethics committee of the University of Mannheim and also approved by the head teachers of the respective schools. Participants with a medical condition or neurological disorders were excluded. All participating subjects were assessed by means of self-rating and two external ratings (by their parents and a psychiatrist specialized in pediatrics) based on ICD-10 as well as DSM-IV and the Development and Well-Being Assessment Interview (DAWBA)^36^.

### Questionnaire

The NEO-Five Factor Inventory (NEO-FFI)^46^ was administered at three measurement occasions (Baseline: 14 years of age; Follow-up 1: 16–17 years of age; Follow-up 2: 19 years of age) to assess personality on the basis of self report. 12 Items describing neuroticism from the 60-item-scale were selected for the present analyses. The NEO-FFI represents a short form of the extensive 240-item NEO-PI-R. Items were assessed on a 5-point-Likert scale from strongly disagree to strongly agree. Four items had to be reverse coded.

### Scanning procedure

Structural MRI was performed on 3 Tesla scanners from three manufacturers (Siemens: 5 sites; Philips: 2 sites; and General Electric: 2 sites). The details of the entire MR protocol are described elsewhere. In this study, we used the T1-weighted images. These high-resolution anatomical MRIs were obtained using a three-dimensional sequence based on the ADNI protocol (http://adni.loni.ucla.edu/research/protocols/mri-protocols/).

### Voxel-based morphometry

In order to evaluate if and how structural data from baseline predict developmental changes at following measurement occasions, we applied whole-brain voxel-wise analyses. In ROI-analyses information concerning potentially influential areas is needed, hence rendering it unsuitable for exploratory analyses. Structural data was preprocessed by means of the VBM8 toolbox (http://dbm.neuro.uni-jena.de/vbm.html) and SPM8 (http://www.fil.ion.ucl.ac.uk/spm) with default parameters. The VBM8 toolbox involves bias correction, tissue classification and affine registration. The affine registered grey matter (GM) and white matter (WM) segmentations were used to build a customized DARTEL (diffeomorphic anatomical registration through exponentiated lie algebra) template. Then warped GM and WM segments were created. Modulation was applied in order to preserve the volume of a particular tissue within a voxel by multiplying voxel values in the segmented images by the Jacobian determinants derived from the spatial normalization step. In effect, the analysis of modulated data tests for regional differences in the absolute amount (volume) of GM/WM. Images were smoothed with a FWHM (full-width at half maximum) kernel of 8 mm^47^.

### Structural equation modelling

We applied structural equation modelling to analyze development of neuroticism on self-reported questionnaire data. After testing different degrees of measurement invariance across time, second-order latent growth curve models were used to model development. Applying second-order latent growth curve models in the present study has the advantage that, first, measurement invariance can be tested for and, second, reliability of the estimation of the slope and intercept is improved (for details on the latter see^48^). Measurement invariance describes the degree of stability of psychometric characteristics of a questionnaire allowing for interpretation of change on latent level without confounding measurement errors or different underlying psychometric properties^49^. Measurement invariance is most often tacitly assumed, for example in applying first-order growth curve models with sum scores as indicator, but rarely explicitly tested. Three degrees of measurement invariance were assessed in the present study. Configural invariance requires the same items to load on the same factor across time, implying that the same items can be assigned to the same theoretical construct across measurement occasions. For weak invariance to hold, factor loadings are constrained to be equal across time, implying that the information contributed by every item to the underlying construct remains the same across time. Strong factorial invariance requires constraining the intercepts to be equal across time. Establishing strong measurement invariance enables interpretation of mean changes on latent level as changes of the latent construct and not changes due to systematic differences on item-level. We used MLR estimator in our analyses (i.e., a maximum likelihood estimator with robust standard errors). As criteria for model fit, we report the Comparative Fit Index (CFI), and the Root Mean Square Error of Approximation (RMSEA). Values of the CFI above 0.95 denote a well-fitting model, whereas for the RMSEA values less than 0.06 may be interpreted as acceptable model fit^50^. In addition, we report adjusted *χ*^*2*^-values, degrees of freedom (*df*), and corresponding p-values for all models examined as well as the Satorra-Bentler scaled *χ*^*2*^-difference-test for comparing nested models^51^. A total of 12 manifest variables from the NEO-FFI served as indicators for the latent neuroticism-factor in a one-factor-model, measured across three time-points. For model identification, effects-coding method was used according to Little *et al*.^52^. As time-invariant covariates measured at baseline, sex, age, scanner-site, puberty stage, depression at baseline and, finally, voxelwise whole-brain MRI-data were added. We chose time-invariant covariates over time-varying covariates as the former are well suited for explaining variation of intercept and slope, whereas the latter rather address occasion-specific variance in the dependent variables, which was not the focus of our paper^53^. Sex was dummy-coded with 1 = female and 0 = male. Age was assessed as days since birth. The nine different scanner-sites were dummy-coded each with Berlin as reference. Puberty was assessed with the Pubertal Development Scale^54^. Depression was extracted from the Development and Well-Being Assessment Interview (DAWBA)^36^.

Throughout the analyses we used R (3.1.1) and Mplus version 8.0^55^.

## Data Availability

The dataset analyzed during the current study is available from the first author Simone Kühn on reasonable request.
